# Seeding for sirtuins: microseed matrix seeding to obtain crystals of human Sirt3 and Sirt2 suitable for soaking

**DOI:** 10.1107/S2053230X15019986

**Published:** 2015-11-18

**Authors:** Tobias Rumpf, Stefan Gerhardt, Oliver Einsle, Manfred Jung

**Affiliations:** a Institute of Pharmaceutical Sciences, Albert-Ludwigs-University Freiburg, Albertstrasse 25, 79104 Freiburg, Baden-Württemberg, Germany; b Institute of Biochemistry and BIOSS Centre for Biological Signalling Studies, Albert-Ludwigs-University Freiburg, Albertstrasse 21, 79104 Freiburg, Baden-Württemberg, Germany

**Keywords:** sirtuins, deacylase, deacetylase, seeding, sirtuin–inhibitor complex

## Abstract

Sirtuins constitute a family of NAD^+^-dependent enzymes that catalyse the cleavage of various acyl groups from the ∊-amino group of lysines. They regulate a series of cellular processes and their misregulation has been implicated in various diseases, making sirtuins attractive drug targets. To date, only a few sirtuin modulators have been reported that are suitable for cellular research and their development has been hampered by a lack of structural information. In this work, microseed matrix seeding (MMS) was used to obtain crystals of human Sirt3 in its apo form and of human Sirt2 in complex with ADP ribose (ADPR). Crystal formation using MMS was predictable, less error-prone and yielded a higher number of crystals per drop than using conventional crystallization screening methods. The crystals were used to solve the crystal structures of apo Sirt3 and of Sirt2 in complex with ADPR at an improved resolution, as well as the crystal structures of Sirt2 in complex with ADPR and the indoles EX527 and CHIC35. These Sirt2–ADPR–indole complexes unexpectedly contain two indole molecules and provide novel insights into selective Sirt2 inhibition. The MMS approach for Sirt2 and Sirt3 may be used as the basis for structure-based optimization of Sirt2/3 inhibitors in the future.

## Abstract 2

In the present study, microseed matrix seeding was successfully applied to obtain a large number of crystals of the human sirtuin isotypes Sirt2 and Sirt3. These crystals appeared predictably in diverse crystallization conditions, diffracted to a higher resolution than reported in the literature and were subsequently used to study the protein–ligand interactions of two indole inhibitors.

## 1. Introduction   

Sirtuins are a unique NAD^+^-dependent enzyme family conserved from bacteria to humans that catalyse the cleavage of acyl groups from the ∊-amino group of lysines (Brachmann *et al.*, 1995 ▸). Originally thought to function only as lysine deacetylases, it was recently reported that sirtuins are also able to cleave off other acyl groups (Feldman *et al.*, 2013 ▸). Sirt5 efficiently converts proteins containing succinylated, malonylated and glutarylated lysines, while the isotypes Sirt1, Sirt2, Sirt3 and Sirt6 preferentially cleave off fatty-acid moieties from the ∊-amino group of lysines (Du *et al.*, 2011 ▸; Bao *et al.*, 2014 ▸; Tan *et al.*, 2014 ▸; Liu *et al.*, 2015 ▸; Teng *et al.*, 2015 ▸). Additionally, several sirtuin isotypes have also been described to be ADP-ribosylases (Du *et al.*, 2009 ▸; Pan *et al.*, 2011 ▸). However, the physiological roles of post-translational modifications other than acetylation are far from being understood and still need to be further investigated. The initial interest in sirtuins was provoked by reports that sirtuins are critical regulators of aging and that overexpression of specific isotypes may prolong lifespan and impede the onset of age-related diseases by imitating the state of calorie restriction (Howitz *et al.*, 2003 ▸; Bordone *et al.*, 2007 ▸). However, extensive research has revealed that sirtuins have many other cellular functions (reviewed in Schemies *et al.*, 2010 ▸). Thus, they are involved in the regulation of cell-cycle control (Vaquero *et al.*, 2007 ▸; Serrano *et al.*, 2013 ▸), metabolism (Hirschey *et al.*, 2010 ▸), stress response (Bell *et al.*, 2011 ▸), autoimmunity (Chuprin *et al.*, 2015 ▸) and mitochondrial biogenesis (Milne *et al.*, 2007 ▸). Their misregulation has been linked to a variety of pathological processes involved in neurodegenerative diseases (Outeiro *et al.*, 2007 ▸) and metabolic disorders (Hirschey *et al.*, 2011 ▸). Generally considered as tumour suppressors (Kim *et al.*, 2010 ▸, 2011 ▸), Sirt1, Sirt2 and Sirt3 have also been shown to assume a role in promoting tumorigenesis in various forms of cancer (Alhazzazi *et al.*, 2011 ▸; Liu *et al.*, 2013 ▸; Yang *et al.*, 2013 ▸). Sirtuins are therefore considered to be potential drug targets. They may also represent targets for antiparasitic drugs (Lancelot *et al.*, 2013 ▸). However, cellular studies as well as animal studies have been hampered by the lack of suitable modulators. Of the seven members of the human sirtuin family, Sirt2 is mainly found in the cytoplasm, while Sirt3 is the main mitochondrial deacylase (North *et al.*, 2003 ▸; Lombard *et al.*, 2007 ▸).

**Figure 1:**
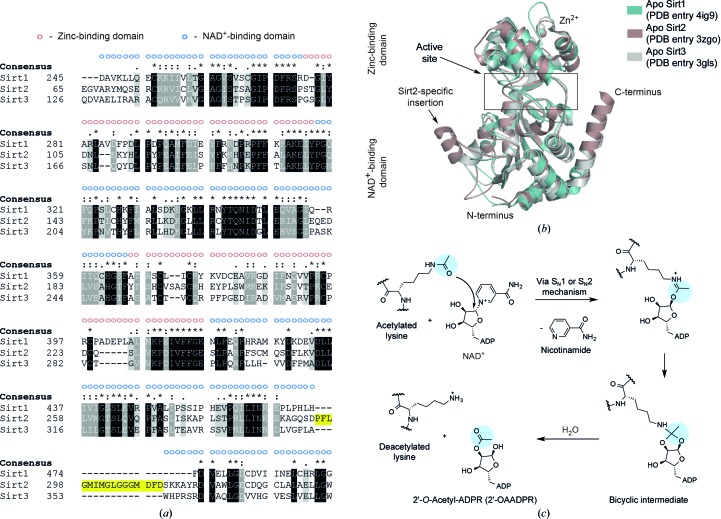
The NAD^+^-dependent deacylases share a highly conserved catalytic core that consists of a larger NAD^+^-binding domain and a smaller zinc-binding domain. (*a*) Structural sequence alignment of the deacylase domains of human Sirt1 (UniProt Q96EB6; amino acids 244–495), human Sirt2 (UniProt Q8IXJ6; amino acids 65–337) and human Sirt3 (UniProt Q9NTG7; amino acids 126–379). The Sirt2-specific insertion is marked in yellow. The deacylase domains of Sirt1, Sirt2 and Sirt3 share a sequence identity of 44%. (*b*) Superposition of the deacylase domains of apo Sirt1 (teal; PDB entry 4ig9), apo Sirt2 (brown; PDB entry 3zgo) and apo Sirt3 (light grey; PDB entry 3gls). The NAD^+^-binding domain of Sirt1, Sirt2 and Sirt3 is very similar, while the zinc-binding domain is structurally more variable. (*c*) Proposed mechanism of sirtuin-catalyzed deacetylation. The carbonyl O atom of the acetyl group attacks the C1′ atom of the ribose. On nicotinamide cleavage, the acetyl group is then transferred *via* an alkylimidate and a bicyclic intermediate to the 2′-­hydroxy group of the ribose. The deacetylated lysine is subsequently released. The final reaction product 2′-*O*-AADPR equilibrates non-enzymatically with 3′-*O*-AADPR. Deacylation is thought to take place using a similar mechanism.

Sirtuins consist of a highly conserved catalytic deacylase domain of around 275 amino acids that is flanked by unstructured N- and C-termini that differ in their length and in their sequence. They have been grouped into four different classes, of which the class I sirtuins Sirt1, Sirt2 and Sirt3 share the highest sequence identity (Fig. 1 ▸ *a*). The crystal structures of the deacylase domain of all sirtuin isotypes except Sirt4 and Sirt7 in differently ligated states have been solved to date (Finnin *et al.*, 2001 ▸; Jin *et al.*, 2009 ▸; Du *et al.*, 2011 ▸; Pan *et al.*, 2011 ▸; Zhao *et al.*, 2013 ▸; Davenport *et al.*, 2014 ▸). They show a two-domain structure consisting of a large Rossmann-fold domain and a smaller zinc-binding domain that is typical of sirtuins (Fig. 1 ▸ *b*). The two domains are separated by a large cleft that constitutes the catalytic core. Both domains are connected *via* a series of loops that play important roles during cofactor binding, acyl-lysine binding and the ‘open-to-closed’ rotation. During catalysis, NAD^+^ adopts a kinked conformation which brings the C1′ of its ribose moiety into proximity for a nucleophilic attack by the carbonyl O atom of the acetyl lysine that is inserted in a hydrophobic tunnel (Fig. 1 ▸ *c*). On cleavage of nicotinamide and the formation of an alkylimidate complex, the acetyl group is transferred *via* several intermediates from the ∊-amino group to the ADP ribose (ADPR) moiety, generating 2′-*O*-acetyl-ADPR and releasing the deacetylated lysine. Deacylation is thought to proceed in a similar fashion (Feldman *et al.*, 2015 ▸). Structural information on sirtuin inhibition is still rather scarce and only recently have several structures of sirtuins in complex with inhibitors been reported (Disch *et al.*, 2013 ▸; Gertz *et al.*, 2013 ▸; Zhao *et al.*, 2013 ▸; Nguyen, Gertz *et al.*, 2013 ▸; Nguyen, Schafer *et al.*, 2013 ▸; Yamagata *et al.*, 2014 ▸; Rumpf *et al.*, 2015 ▸). Additional work is still needed to develop rationales for the design of potent and selective modulators that are suitable for cellular studies.

Crystallization of proteins often turns out to be a very demanding objective and initial crystals usually require optimization for diffraction experiments. Seeding is one of the methods that has helped to facilitate the optimization process (Stura & Wilson, 1991 ▸; Bergfors, 2003 ▸; D’Arcy *et al.*, 2003 ▸). It has been shown to enhance the reproducibility of crystal formation and to improve crystal morphology as well as diffraction. Ireton and Stoddard extended the concept of seeding to what they termed microseed matrix seeding (MMS), in which microseeds are transferred to a matrix of diverse buffer conditions (Ireton & Stoddard, 2004 ▸). D’Arcy and coworkers incorporated the addition of microseed solutions to screening procedures with standard crystallization robots (D’Arcy *et al.*, 2007 ▸). MMS is also widely used in drug-discovery programs. It is usually the best way to obtain large numbers of crystals for high-throughput soaking experiments. Cross-seeding, an approach in which seed crystals of one homologue are used to initiate crystal formation of another one, has also been applied successfully (Obmolova *et al.*, 2010 ▸). Current developments in the field of MMS have recently been reviewed and summarized (D’Arcy *et al.*, 2014 ▸).

In this work, we describe for the first time the successful application of MMS techniques to human isotypes Sirt2 and Sirt3 from the sirtuin family. We obtained well diffracting crystals of Sirt3 in its apo form and of Sirt2 in complex with the product analogue ADP ribose (ADPR) in diverse crystallization conditions. Using MMS, crystal formation was predictable, less error-prone and yielded a large number of crystals. The crystals were used to obtain crystal structures of Sirt2 in complex with ADPR and of apo Sirt3 at an improved resolution. They also proved to be perfectly suited for the investigation of protein–ligand interactions and were subsequently used to solve two novel crystal structures of Sirt2 in complex with ADPR and indole inhibitors. Both Sirt2–ADPR–indole complexes unexpectedly contained two inhibitor molecules in the active site of Sirt2, highlighting the specific characteristics of Sirt2 within the sirtuin family.

## 2. Materials and methods   

### 2.1. Cloning, protein expression and protein purification   

The gene sequences encoding human Sirt2 (UniProt Q8IXJ6; amino acids 50–356 or 56–356) and human Sirt3 (UniProt Q9NTG7; amino acids 118–395) were cloned in a modified pET-15b vector with a His_10_ tag instead of a His_6_ tag and a TEV protease cleavage site instead of a thrombin cleavage site. Human Sirt2_50–356_, Sirt2_56–356_ and Sirt3_118–395_ were expressed in *Escherichia coli* strain BL21(DE3) CodonPlus RIPL cells overnight (20°C for Sirt2 and 18°C for Sirt3). Overexpression was induced with IPTG (0.1 m*M*) at an OD_600_ of 0.6. The cells were harvested and resuspended in lysis buffer [50 m*M* Tris–HCl, 500 m*M* NaCl, 5%(*v*/*v*) glycerol, 5 m*M* β-mercaptoethanol pH 8.0 for Sirt2_56–356_; 50 m*M* HEPES, 500 m*M* NaCl, 5%(*v*/*v*) glycerol, 5 m*M* β-mercapto­ethanol pH 7.5 for Sirt3 and Sirt2_50–356_]. The cells were then lysed with a microfluidizer (Microfluidics, Westwood, USA) and cell debris was removed *via* centrifugation. The supernatant was applied onto a HisTrap FF column (5 ml; GE Healthcare, Freiburg, Germany), washed intensively with lysis buffer and then treated with TEV protease (in excess). After overnight digestion (4°C), the digested protein was eluted with lysis buffer, concentrated and further purified with a Superdex S75 26/60 gel-filtration column [GE Healthcare; 25 m*M* Tris–HCl, 150 m*M* NaCl pH 8.0 for Sirt2_56–356_; 25 m*M* HEPES, 200 m*M* NaCl, 5%(*v*/*v*) glycerol, 5 m*M* β-mercapto­ethanol pH 7.5 for Sirt3 and Sirt2_50–356_]. Sirt2- or Sirt3-containing fractions were collected and concentrated to 20 mg ml^−1^ in the case of Sirt2_50–356_, 13 mg ml^−1^ in the case of Sirt2_56–356_ and 18.5 mg ml^−1^ in the case of Sirt3. All purification steps were monitored by SDS–PAGE (Laemmli, 1970 ▸) and the protein concentration was determined by the Bradford assay (Bradford, 1976 ▸).

### 2.2. Crystallization and soaking experiments   

All crystallization trials were performed in 96-well plates (Intelli-Plate 96-3 Low Profile, Art Robbins Instruments, Sunnyvale, USA) using an OryxNano pipetting robot (Douglas Instruments, Berkshire, England). Index screen was obtained from Hampton Research (Aliso Veijo, USA). The composition of the crystallization solutions in the Index screen can be found at http://hamptonresearch.com/documents/product/hr005585_2-134_formulations.pdf. Crystal formation was monitored with a Minstrel HT UV imaging unit (Rigaku, Kent, England). Initial crystals that were used for MMS of apo Sirt3 (18.5 mg ml^−1^) were obtained in a solution consisting of 0.2 *M* Li_2_SO_4_, 60%(*v*/*v*) Tacsimate (Hampton Research) at pH 7.0 and 4°C with a 1:1 ratio of Sirt3 solution to reservoir solution. Initial crystals of the Sirt2_56–356_–ADPR complex (13 mg ml^−1^, 20 m*M* ADPR from a 1 *M* stock solution in 1 *M* Tris–HCl buffer pH 9.0) were obtained in a solution consisting of 17.5%(*w*/*v*) PEG 10 000, 0.1 *M* ammonium acetate in 0.1 *M* bis-tris buffer pH 6.75 at 20°C using a 1:3 ratio of Sirt2–ADPR solution to reservoir solution. Microseed solutions were prepared as follows: 5–10 crystals were harvested, washed, diluted with mother liquor and transferred into an Eppendorf tube, where they were crushed with a seed bead [five cycles of slight vortexing (10 s) followed by incubation on ice (20 s)]. The supernatant was then used for crystallization trials. For crystallization trials using microseed solutions the drop consisted of 17%(*v*/*v*) microseed solution, 50–33%(*v*/*v*) Sirt3 solution (18.5 mg ml^−1^) or Sirt2–ADPR solution (13 mg ml^−1^, 20 m*M* ADPR) and 33–50%(*v*/*v*) reservoir solution. Control experiments to verify that Sirt3 crystal formation was dependent on microseeds were performed with the mother liquor only [60%(*v*/*v*) Tacsimate pH 7.0 or 60%(*v*/*v*) Tacsimate, 0.2 *M* Li_2_SO_4_ pH 7.0].

Crystals of Sirt2_50–356_ in complex with ADPR [20 mg ml^−1^, 10 m*M* NAD^+^ (Sigma–Aldrich, Deisenhofen, Germany), 100 m*M* stock solution in 25 m*M* HEPES, 200 m*M* NaCl, 5%(*v*/*v*) glycerol, 5 m*M* β-mercaptoethanol pH 7.5] were obtained in a solution consisting of 30%(*w*/*v*) PEG 3350, 0.2 *M* NaCl in 0.1 *M* bis-tris buffer pH 6.25 at 4°C. The crystals formed after 3–4 d and were mounted on nylon loops before flash-cooling in liquid nitrogen. Apo Sirt3 crystals were obtained by MMS in a solution consisting of 25%(*w*/*v*) PEG 3350, 0.2 *M* MgCl_2_ in 0.1 *M* bis-tris buffer pH 5.5 at 4°C. The crystals were cryoprotected by the addition of PEG 3350 to a final concentration of 30%(*w*/*v*) PEG 3350, mounted on nylon loops and flash-cooled in liquid nitrogen.

For soaking experiments with indole inhibitors, crystals of Sirt2_56–356_ in complex with ADPR were obtained *via* MMS in a solution consisting of 18%(*w*/*v*) PEG 10 000 in 0.1 *M* bis-tris buffer pH 5.75 at 20°C. These crystals formed after 1 d and were then soaked in a buffer consisting of 18%(*w*/*v*) PEG 10 000, 10%(*v*/*v*) DMSO, 0.1 *M* bis-tris buffer pH 5.75 and either 10 m*M* EX527 (Sigma–Aldrich, racemic) or 10 m*M* CHIC35 (Sigma–Aldrich) for 30–90 min. The crystals were cryoprotected by the addition of 20%(*v*/*v*) glycerol and were then mounted on nylon loops before flash-cooling in liquid nitrogen.

### 2.3. Data collection   

Data were collected on beamlines X06SA (Sirt2_50–356_–ADPR) and X06DA (apo Sirt3) at the Swiss Light Source, Villigen, Switzerland using Pilatus detectors (Dectris, Baden, Switzerland) at 100 K with oscillations of 0.2 or 0.5° and an X-ray wavelength of 1.0 Å. Data for Sirt2_56–356_ in complex with ADPR and EX243 and data for Sirt2_56–356_ in complex with ADPR and CHIC35 were collected using a MicroMax-007 HF rotating-anode X-ray generator (Rigaku) at a wavelength of 1.5418 Å equipped with a MAR345 image-plate detector (MAR Research, Hamburg, Germany). For each structure, one single crystal was used. Data were processed with *MOSFLM* (Leslie & Powell, 2007 ▸) or *XDS* (Kabsch, 2010 ▸) and scaled using the CC_1/2_ criterion (Karplus & Diederichs, 2012 ▸) with *AIMLESS* (Evans & Murshudov, 2013 ▸) from the *CCP*4 suite (Winn *et al.*, 2011 ▸).

### 2.4. Structure solution and refinement   

All structures were solved by molecular replacement with *MOLREP* (Vagin & Teplyakov, 2010 ▸) using a monomer of apo Sirt3 (PDB entry 3gls; Jin *et al.*, 2009 ▸) or a monomer of the Sirt2–ADPR complex (PDB entry 3zgv; Moniot *et al.*, 2013 ▸) as a search model. Model building was carried out with *Coot* (Emsley *et al.*, 2010 ▸) and the structures were refined with *REFMAC*5 (Murshudov *et al.*, 2011 ▸) or *PHENIX* (Adams *et al.*, 2010 ▸). Crystal twinning as well as the twin fractions were detected using *phenix.xtriage* from the *PHENIX* suite (Adams *et al.*, 2010 ▸). Twin refinement was achieved with the intensity-based twin-refinement option of *REFMAC*5. Ligands were generated with the *Grade* web server (Global Phasing Ltd, Cambridge, England) and were placed into 2*F*_o_ − *F*_c_ electron-density maps using *AFITT-CL* (v.2.1.0; OpenEye Scientific Software, Santa Fe, USA). All residues of Sirt2 and Sirt3 except those of the flexible N- and C-termini are defined by electron density. In the crystal structures of the Sirt2–ADPR–indole complexes, a methionine and a histidine are seen at the N-terminus. They originate from the NdeI restriction site of the pET vector. All structures were validated using *PROCHECK* (Laskowski *et al.*, 1993 ▸) and the *MolProbity* server (Chen *et al.*, 2010 ▸). Ramachandran plots for each structure are shown in Supplementary Fig. S3. The data-collection and refinement statistics are summarized in Table 1 ▸. The structural sequence alignment was generated with *T-Coffee* (Di Tommaso *et al.*, 2011 ▸) and the images were prepared with *PyMOL* (v.1.6; Schrödinger). Iterative-build OMIT electron-density maps for the ligands of all structures as well as the hinge loop of the Sirt2–ADPR complex and the surface of the binding pockets were generated with the *PHENIX* suite (Terwilliger *et al.*, 2008 ▸; Adams *et al.*, 2010 ▸) and *HOLLOW* (Ho & Gruswitz, 2008 ▸).

## 3. Results   

### 3.1. Seeding   

**Figure 2:**
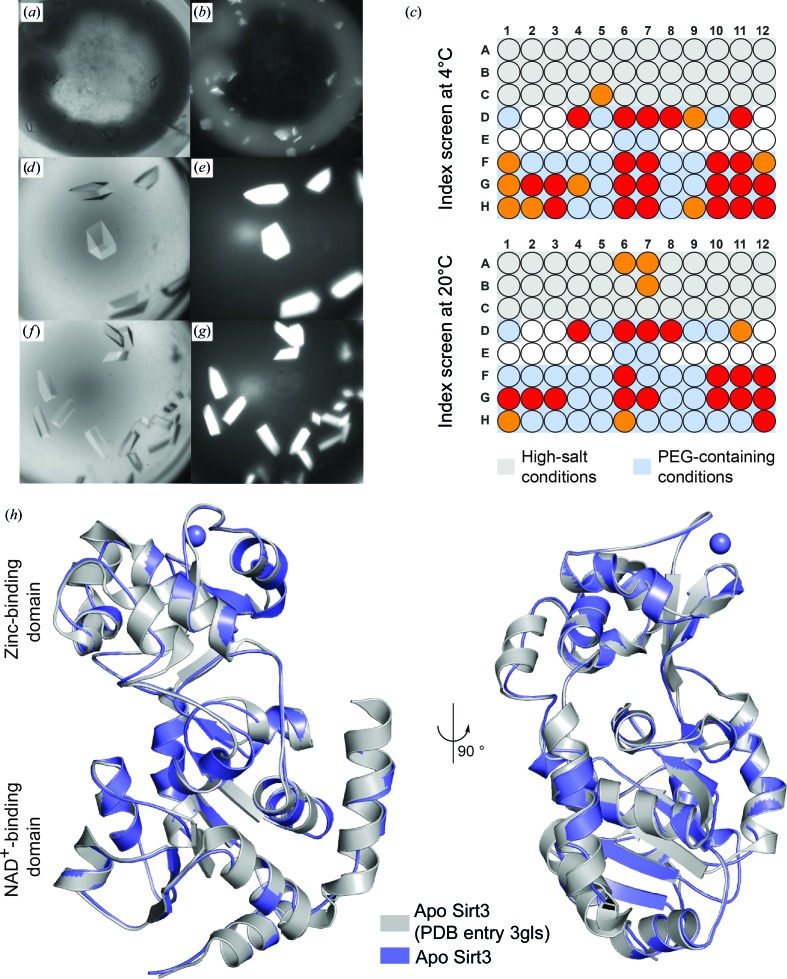
The use of microseeds improves apo Sirt3 crystallization. (*a*) Representative initial apo Sirt3 crystals obtained in a solution consisting of 60%(*v*/*v*) Tacsimate, 0.2 *M* lithium sulfate at 4°C after a week. (*b*) UV image of (*a*). (*c*) Schematic representation of crystal formation in the Index screen (Hampton Research) using MMS with two different crystallization-drop compositions at two different temperatures. A red-coloured circle indicates crystal formation in both drops, while an orange circle indicates crystal formation in only one drop. A colourless condition represents no crystal formation. Conditions containing high salt concentrations are marked in light grey and crystallization conditions with more than 10%(*w*/*v*) PEG are marked in light blue. Apo Sirt3 crystallizes in mainly PEG 3350-containing conditions preferentially under acidic conditions at 4°C as well as 20°C. (*d*) Representative crystals of apo Sirt3 obtained with microseeds in Index screen condition D11 [0.1 *M* bis-tris buffer, 28%(*w*/*v*) PEG MME 2000 pH 6.5] at 4°C. (*e*) UV image of (*d*). (*f*) Representative apo Sirt3 crystals obtained with microseeds in Index screen condition G6 [0.2 *M* ammonium acetate, 0.1 *M* bis-tris buffer, 25%(*w*/*v*) PEG 3350 pH 5.5]. (*g*) UV image of (*f*). (*h*) Superposition of apo Sirt3 (PDB entry 3gls, chain *A*, light grey) with the improved apo Sirt3 structure (chain *A*, slate blue). Both structures feature the two-domain structure typical of sirtuins and are very similar (r.m.s.d. of 0.25 Å for all C^α^ atoms).

Initial crystals of apo Sirt3 were obtained in a solution consisting of 0.2 *M* lithium sulfate, 60%(*v*/*v*) Tacsimate at 4°C (Figs. 2 ▸ *a* and 2 ▸ *b*). The epitaxically twinned crystals appeared after one week and diffracted to 2.4–3.0 Å resolution. Further optimization of the crystallization conditions did not improve either the crystal morphology or diffraction. Furthermore, initial soaking experiments failed owing to the insolubility of our drug-like ligands in the highly polar Tacsimate condition. We therefore opted for seeding. The use of microseeds in the Index screen (Hampton Research), as described by D’Arcy *et al.* (2007 ▸), proved to be very successful. In the presence of the microseeds, apo Sirt3 crystallized rapidly at different temperatures, in diverse crystallization conditions and in a pH range from 3.5 to 8.5 (Fig. 2 ▸ *c*). The best results were obtained with a microseed solution of 1–2 crystals per 10 µl reservoir solution. In most cases, mainly in acidic PEG 3350-containing conditions, apo Sirt3 formed single crystals (Figs. 2 ▸ *d*, 2 ▸ *e*, 2 ▸ *f* and 2 ▸ *g*) predictably within several days. They showed an improved diffraction pattern to a resolution of below 2.0 Å. During the initial crystallization screenings we also found that the presence of lithium sulfate was an important nucleation factor, as the initial crystals could only be obtained in the presence of lithium sulfate. We also investigated the influence of lithium sulfate on the crystallization of apo Sirt3 in the presence of microseeds using the Index screen (Hampton Research). Again, apo Sirt3 crystallized rapidly in diverse conditions, over a wide pH range and at different temperatures within days (Supplementary Fig. S1). In a control experiment, we also performed crystallization trials in a similar fashion with the same seeding solutions but without microseeds. Here, we could only observe crystal formation in two different crystallization conditions (data not shown). The formation of the apo Sirt3 crystals was therefore dependent on the presence of microseeds.

### 3.2. Crystal structure of human apo Sirt3   

Diffraction data from a crystal obtained using microseeds were used to solve the structure of the apo form of human Sirt3 by molecular replacement at a resolution of 1.83 Å [the resolution of the crystal structure of apo Sirt3 deposited by Jin *et al.* (2009 ▸) with PDB code 3gls is 2.7 Å]. Sirt3 crystallized in space group *P*2_1_ with six monomers in the asymmetric unit. All monomers feature the typical sirtuin-like two-domain structure with a larger Rossmann-fold domain and a smaller zinc-binding domain and adopt the ‘open’ conformation. However, they do show some differences within the asymmetric unit (r.m.s.d. ranging from 0.2 to 0.8 Å for all C^α^ atoms of each monomer). A similar behaviour has also been observed in the published crystal structure of human apo Sirt3 (Jin *et al.*, 2009 ▸). Superposition of chain *A* of the published apo Sirt3 structure (PDB entry 3gls) with chain *A* of the improved structure of Sirt3 shows a high similarity (r.m.s.d. of 0.25 Å for all C^α^ atoms). Similar to the published X-ray structure of apo Sirt3, the acyl-lysine binding site of all monomers is occupied by a PEG molecule. This has also been observed in other sirtuin structures (Avalos *et al.*, 2004 ▸, 2005 ▸).

### 3.3. Improved crystal structure of human Sirt2 in complex with ADP ribose   

**Figure 3:**
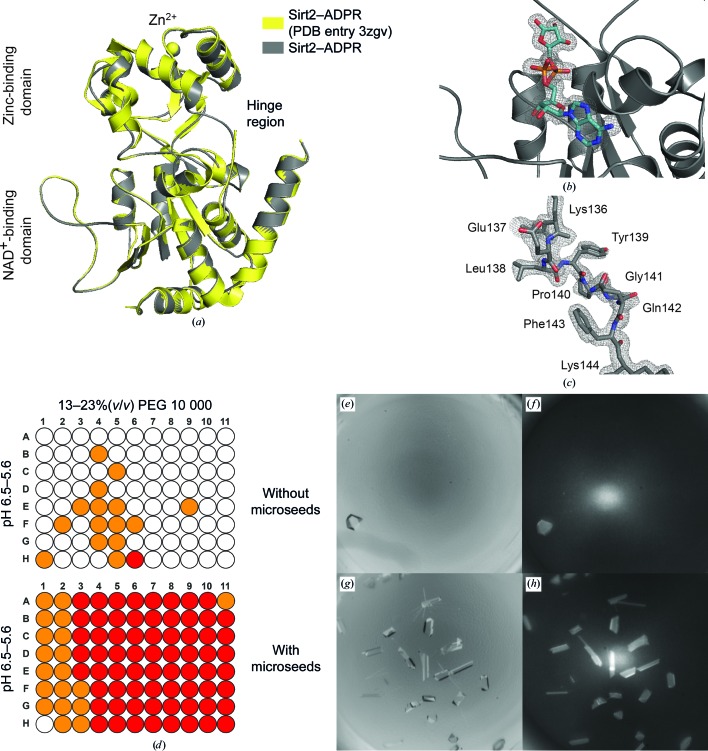
Improved crystal structure of human Sirt2 in complex with the product analogue ADPR. (*a*) Superposition of the published X-ray structure of human Sirt2 in complex with ADPR (PDB entry 3zgv, chain *A*, yellow) with the improved X-ray structure of the Sirt2–ADPR complex (chain *A*, dark grey) shows that the structures are very similar (r.m.s.d. of 0.18 Å for all C^α^ atoms). (*b*) Close-up view of the active site of the improved Sirt2–ADPR complex. ADPR (overall *B* factor of 13.8 Å^2^) is shown as aquamarine sticks and the σ-weighted 2*F*_o_ − *F*_c_ electron-density map is contoured at 1.0σ. A σ-weighted *F*_o_ − *F*_c_ electron-density OMIT map of ADPR is shown in Supplementary Fig. S4. (*c*) Close-up view of the hinge loop of Sirt2. In the published X-ray structure of Sirt2 parts of this loop were not defined by the electron density. In the improved structure of Sirt2, the conformations of all amino acids that form this loop are well defined by the electron density. The σ-weighted 2*F*_o_ − *F*_c_ electron-density map is also contoured at 1.0σ. A σ-weighted *F*_o_ − *F*_c_ electron-density OMIT map of the hinge loop is shown in Supplementary Fig. S4. (*d*) Schematic representation of crystal hits for the complex of human Sirt2_56–356_ and ADPR in a screen to optimize the initial crystallization conditions. Two different crystallization-drop compositions were used. In the presence of microseeds, Sirt2_56–356_ crystallizes more rapidly and yields more crystals using diverse drop compositions, pH and PEG 10 000 concentrations. A red condition indicates crystals in both drops, while orange indicates crystal formation in only one. (*e*) Representative Sirt2–ADPR crystal after 5 d in a solution consisting of 15%(*w*/*v*) PEG 10 000 in 0.1 *M* bis-tris buffer pH 6.0. (*f*) UV image of (*e*). (*g*) Representative crystals of the Sirt2–ADPR complex obtained in the presence of microseeds in the solution mentioned in (*e*). (*h*) UV image of (*g*).

We used two different truncated forms of Sirt2 (Sirt2_50–356_ and Sirt2_56–356_) for crystallization trials of Sirt2. Cross-seeding using microseed solutions of apo Sirt3 failed. However, we succeeded using conventional screening methods and obtained crystals of both Sirt2 forms in complex with ADPR. Initially, Sirt2_50–356_ crystallized in the presence of the cosubstrate NAD^+^ (2 m*M*) after a month in Index screen condition F10 (Hampton Research) with a 1:1 ratio of reservoir solution to protein solution at 4°C. This crystallization condition was optimized to 30%(*w*/*v*) PEG 3350, 0.2 *M* NaCl in 0.1 *M* bis-tris buffer at pH 6.25 and 4°C. Additionally, the NAD^+^ concentration and the protein:reservoir ratio were increased to 20 m*M* and 3:1, respectively. Thus, Sirt2_50–356_ crystals appeared within 3–4 d. Using the diffraction data obtained from one of these crystals, we determined the space group as *P*2_1_2_1_2_1_ and were able to solve the structure of Sirt2 by molecular replacement at a resolution of 1.63 Å (Figs. 3 ▸ *a* and 3 ▸ *b*; the resolution of the deposited crystal structure of Sirt2–ADPR was 2.27 Å; PDB entry 3zgv; Moniot *et al.*, 2013 ▸). Each Sirt2 molecule contains the product analogue ADPR formed through the hydrolysis of NAD^+^. The structure of Sirt2_50–356_ in complex with ADPR is very similar to the structure of Sirt2–ADPR recently published by Moniot *et al.* (2013 ▸), but everything is observed in greater detail. The asymmetric unit also contains two very similar monomers (r.m.s.d. of 0.19 Å for all C^α^ atoms) that adopt the ‘closed’ sirtuin conformation. In contrast to the recently published structure, we were also able to include all of the residues of one loop of the hinge region (amino acids 136–144) that connects the Rossmann-fold domain to the zinc-binding domain in our model (Fig. 3 ▸ *c*).

However, the handling of the Sirt2_50–356_ crystals turned out to be difficult and the crystals obtained were pseudo-merohedrally twinned. We were also able to obtain crystals with the other truncated form of Sirt2_56–356_ in the presence of ADPR. These crystals formed after just 1 d in an acidic solution of PEG 10 000 at 20°C. They crystallized in the same space group, diffracted equally well, were easier to handle and were not twinned. We also performed MMS with these crystals. As observed for apo Sirt3, the use of microseeds significantly improved the crystallization process in either fine screens or initial screens such as Index screen (Hampton Research; see Figs. 3 ▸ *d*, 3 ▸ *e*, 3 ▸ *f*, 3 ▸ *g* and 3 ▸ *h* and Supplementary Fig. S2). Again, the crystal count and the predictability of crystal formation were better compared with conventional screening methods set up in the absence of microseeds, while the diffraction quality was equally good.

### 3.4. Crystal structures of Sirt2 in complex with ADPR and the indole inhibitors EX243 and CHIC35   

**Figure 4:**
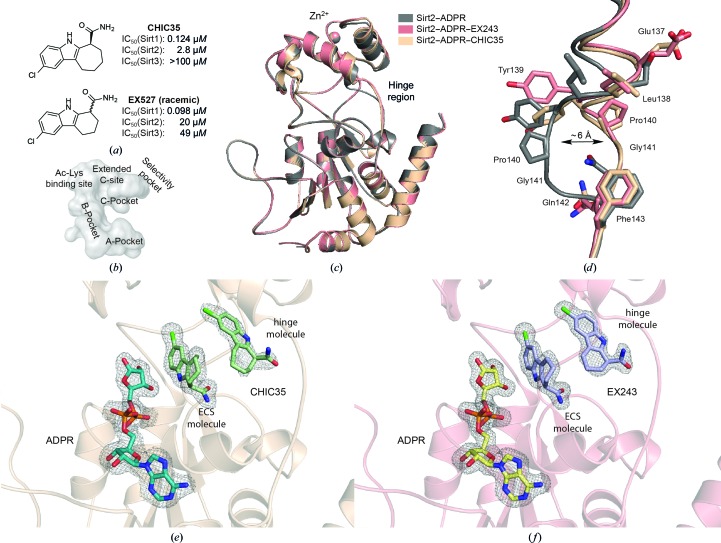
The indole inhibitors CHIC35 and EX243 occupy the extended C-site (ECS) as well as the selectivity pocket with two molecules and induce a conformational change at the hinge region. (*a*) Chemical structures of CHIC35 and EX527 (racemic) and their IC_50_ values to inhibit deacetylation by Sirt1, Sirt2 and Sirt3 (taken from Napper *et al.*, 2005 ▸). The *S*-enantiomer of EX527 is termed EX243. (*b*) Surface representation of the catalytic core of Sirt2 in complex with ADPR. The corresponding subpockets of the catalytic core are labelled according to the literature. (*c*) An overlay of the improved structure of Sirt2–ADPR (grey), the Sirt2–ADPR–EX243 complex (light pink) and the Sirt2–ADPR–CHIC35 complex (salmon) reveals only minor differences in the overall structure; however, significant differences can be observed at the hinge region. (*d*) Close-up view of the hinge region of the structures shown in (*c*). The binding of the two indole molecules induces a 6 Å shift of one hinge loop. (*e*, *f*) Close-up view using the same orientation as shown in (*c*) of the active site of the Sirt2–ADPR–CHIC35 complex (*e*) and the Sirt2–ADPR–EX243 complex (*f*). ADPR is shown as turquoise (Sirt2–ADPR–CHIC35) or light yellow (Sirt2–ADPR–EX243) sticks. CHIC35 is shown as pale green sticks and EX243 as light blue sticks. To better differentiate between the two indole molecules, they are termed the ECS molecule and the hinge molecule, respectively. The σ-weighted 2*F*_o_ − *F*_c_ electron-density map is contoured at 1.0σ. The cofactor-binding loop is not shown for the sake of clarity. σ-Weighted *F*_o_ − *F*_c_ electron-density OMIT maps for ADPR and the indole inhibitors are shown in Supplementary Figs. S4 and S5.

Owing to the lack of potent Sirt3 modulators, we focused our studies on Sirt2 to validate the suitability of the MMS crystals for soaking experiments. Napper and coworkers published a set of indole inhibitors that show a preference for inhibiting Sirt1 but also block Sirt2 and Sirt3 (Napper *et al.*, 2005 ▸; Gertz *et al.*, 2013 ▸). Of these indoles, EX527 (selisistat) and CHIC35 are the most potent inhibitors (Fig. 4 ▸ *a*). Both have been widely used to study sirtuins *in vivo* (Solomon *et al.*, 2005 ▸; Smith *et al.*, 2014 ▸), and EX527 is the first sirtuin inhibitor to date that has been evaluated in clinical trials for the treatment of Huntington’s disease (Süssmuth *et al.*, 2015 ▸; Westerberg *et al.*, 2015 ▸). Furthermore, the indole inhibitors are some of the few inhibitors that have been cocrystallized with human Sirt1, human Sirt3 and the archaeal sirtuin Sir2Tm (Gertz *et al.*, 2013 ▸; Zhao *et al.*, 2013 ▸). The crystal structures revealed that the more potent *S*-enantiomer of the indoles is bound to the active site of Sirt1 and Sirt3. The carboxamide of the indole occupies the C-pocket and mimics the physiological inhibitor nicotinamide (Fig. 4 ▸ *b*). The chlorinated hydrophobic indole moiety also extends into another binding pocket adjacent to the C-pocket that has been termed the extended C-site (ECS). In addition to the indole, all sirtuin–indole complexes contain either the cofactor NAD^+^ or the product analogue ADPR. This is in line with kinetic studies, which concluded that the presence of NAD^+^ or the product analogue ADPR is essential for binding of the indole inhibitor (Napper *et al.*, 2005 ▸; Gertz *et al.*, 2013 ▸; Zhao *et al.*, 2013 ▸). We therefore assumed that the Sirt2–ADPR crystals were perfectly suited for soaking experiments with the indole inhibitors.

**Figure 5:**
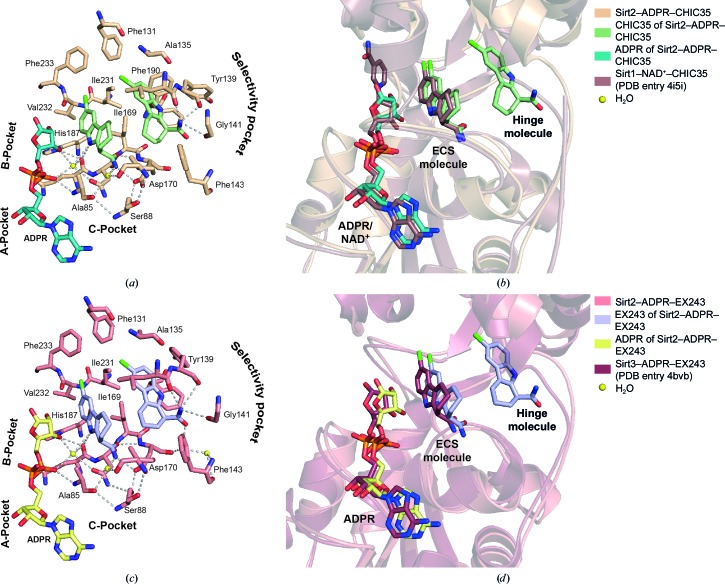
The indole inhibitors CHIC35 and EX243 interact with the residues of the active site of Sirt2 in a similar fashion as observed in other sirtuin–indole complexes. (*a*) The residues that interact with the two molecules of CHIC35 (*a*) or EX243 (*c*) are shown as sticks. Asn168 and Ile169, which are located beneath the ECS molecules, are not labelled. Pro94, Phe96, Leu103, Phe119, Leu134 and Leu138 are not shown for the sake of clarity. The carboxamide moiety of the ECS molecule of both Sirt2–ADPR–indole complexes hydrogen-bonds to the highly conserved residues Asp170 and Ile169 and, *via* a structural water, to Ala85, Ile93 and Pro94 (not shown). For both inhibitors, the amide of the ECS molecule interacts with Gln169 and, *via* another structural water, with ADPR and Asn168. The aromatic chlorinated indole protrudes into the hydrophobic extended C-site. The binding of the hinge molecule is mainly driven by hydrophobic interactions with the side chains of Leu103, Phe119, Phe131, Ala135, Leu138 (not shown), Tyr139 and Phe190. Additionally, the carboxamide moiety of the hinge molecule also hydrogen-bonds to the backbone carbonyl O atom of Leu138 (not shown), Tyr139, Gly141 and, *via* a structural water, to Asp170. Waters are shown as yellow spheres and hydrogen bonds are shown as grey dashes. (*b*, *d*) The binding mode of the ECS molecule of both Sirt2–ADPR–indole complexes is very similar to that observed in the analogous complexes of Sirt1 (brown, PDB entry 4i5i) or Sirt3 (ruby, PDB entry 4bvb).

Soaking with both indole inhibitors proved to be successful, and DMSO concentrations of up to 10%(*v*/*v*) with soaking durations of 90 min did not deteriorate the diffraction pattern of the crystals. Using the diffraction data obtained from the soaked crystals, we were able to solve the crystal structures of Sirt2 in complex with ADPR and either EX243 (EX243 is the *S*-enantiomer of EX527; Sirt2–ADPR–EX243 structure) or CHIC35 (Sirt2–ADPR–CHIC35 structure) by molecular replacement. The space group of the soaked crystals was determined to be *P*2_1_2_1_2_1_, similar to the space group of the unsoaked Sirt2–ADPR crystals. The asymmetric unit contains two monomers that show no significant differences [r.m.s.d.s of 0.29 Å (Sirt2–ADPR–EX243) and 0.23 Å (Sirt2–ADPR–CHIC35) for all C^α^ atoms]. The following descriptions are therefore based on chain *A*. The Sirt2–ADPR–indole complexes adopt the ‘closed’ conformation and share a very close resemblance to the Sirt2–ADPR structure described in §[Sec sec3.3]3.3 [r.m.s.d.s of 0.23 Å (Sirt2–ADPR–EX243) and 0.14 Å (Sirt2–ADPR–CHIC35) for all C^α^ atoms; Fig. 4 ▸ *c*]. The only significant differences can be observed at the hinge region (Fig. 4 ▸ *d*). Here, the indole inhibitors induce a 6 Å shift of one hinge loop (amino acids 136–144; Fig. 4 ▸ *d*). The ADPR molecules of both structures assume a position that is almost identical to that observed in the Sirt2–ADPR complex. To our surprise, each Sirt2 molecule of both Sirt2–ADPR–indole complexes contains two identical indole molecules in an *S*-configuration (Figs. 4 ▸ *e* and 4 ▸ *f*). One molecule occupies the C-pocket as well as the extended C-site, and we will refer to this molecule as the ‘ECS molecule’. The other molecule is found at the hinge region in a pocket that we recently described as the selectivity pocket (Rumpf *et al.*, 2015 ▸). We will refer to this molecule as the ‘hinge molecule’. Both indole molecules of each structure are well defined by electron density; however, the higher *B* factors of the hinge molecule indicate that the hinge molecule is not present in all Sirt2 molecules or that it is more mobile within the selectivity pocket of Sirt2 (*e.g.* *B* factors of 24.1 Å^2^ for the ECS molecule of Sirt2–ADPR–EX243 and 45.6 Å^2^ for the hinge molecule of Sirt2–ADPR–EX243). The ECS molecule in both Sirt2–ADPR–indole complexes is involved in a network of hydrophilic and hydrophobic interactions with Sirt2, ADPR and two highly coordinated water molecules (Figs. 5 ▸ *a* and 5 ▸ *c*). The carboxamide of both indole inhibitors binds in a similar fashion as the carboxamide moiety of the physiological inhibitor nicotinamide and hydrogen-bonds to the highly conserved residues Ile169 and Asp170 and, *via* one of the water molecules (W24 in Sirt2–ADPR–CHIC35 and W206 in Sirt2–ADPR–EX243), to Ala85, Ile93 and Pro94. The amide of the indole hydrogen-bonds to Gln167 and, *via* the other highly coordinated water (W3 in Sirt2–ADPR–CHIC35 and W203 in Sirt2–ADPR–EX243), to Asp168 and ADPR. The ECS molecule also interacts with the hydrophobic side chains of Ile93 and Phe96 as well as the hinge molecule. The binding of the hinge molecule is mainly driven by hydrophobic inter­actions with the side chains of Ala135, Leu138, Tyr139, Phe143 and Phe190. Additionally, the carboxamide moiety of the hinge molecule forms hydrogen bonds to the backbone carbonyl O atoms of Leu138, Tyr139 and Gly141. The interaction pattern of both indole inhibitors in the Sirt2–ADPR–indole complexes is nearly identical. Slight differences can be observed for the conformation of the side chains of Tyr139 and Phe190. Similar interaction patterns for the ECS molecule of both Sirt2–ADPR–indole complexes have also been observed in the crystal structures of Sirt1 and Sirt3 (Gertz *et al.*, 2013 ▸; Zhao *et al.*, 2013 ▸).

## 4. Discussion and conclusions   

In the presented work, we used an MMS approach to obtain crystals of the potential drug targets human Sirt2 and human Sirt3. With microseeds, we were able to obtain large numbers of crystals predictably in solutions of versatile compositions. Crystal formation was not limited to a specific pH and was not dependent on the presence of a specific crystallization-condition component. As we sought to use these crystals of Sirt2 and Sirt3 for structured-based inhibitor-development studies, the MMS approach provided diverse crystallization conditions to perform soaking experiments. This would not have been possible with such ease using conventional screening approaches. The MMS crystals also diffracted to a higher resolution or equally well in comparison to crystals obtained by conventional screening methods. Using these MMS apo Sirt3 crystals, we were able to solve the X-ray structure of apo Sirt3 at a higher resolution than in the deposited X-ray structure. Additionally, we also provide an improved crystal structure of the Sirt2–ADPR complex.

**Figure 6:**
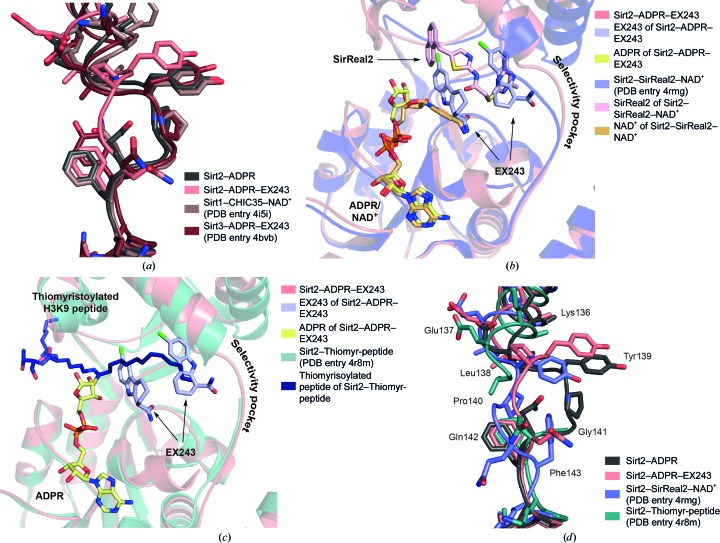
One of the hinge loops of Sirt2 exhibits a high flexibility and seems to be important for inhibitor binding. (*a*) Superposition of the hinge loops of Sirt1, Sirt2 and Sirt3 in complex with either EX527 or CHIC35 (Sirt1, brown cartoon; Sirt2, deep salmon; Sirt3, ruby) and of the hinge loop of Sirt2–ADPR lacking an indole (dark grey). Only the hinge loop of the Sirt2–ADPR–EX243 complex adopts a different conformation, while the hinge-loop conformation of the complexes of Sirt1, Sirt2 and Sirt3 with indole is similar to that observed in the Sirt2–ADPR complex. This loop shift is induced by the binding of the hinge molecule. (*b*) Superposition of the Sirt2–SirReal2–NAD^+^ complex structure (PDB entry 4rmg; slate blue cartoon with SirReal2 in light pink sticks and NAD^+^ in light orange sticks) with the crystal structure of Sirt2–ADPR–EX243 (deep salmon cartoon with ADPR in yellow sticks and EX243 in light blue sticks) reveals that the hinge molecule of the Sirt2–ADPR–EX243 complex occupies the selectivity pocket that is occupied by the dimethylpyrimidine moiety (DMP) in the Sirt2–SirReal2 complex. (*c*) Superposition of the Sirt2–thiomyristoylated peptide complex structure (Sirt2–Thiomyr-peptide; PDB entry 4r8m; turquoise cartoon with the thiomyristoylated peptide shown as dark blue sticks) with the crystal structure of Sirt2–ADPR–EX243 shows that the hydrophobic myristoyl moiety also occupies the selectivity pocket that is occupied by the hinge molecule in the Sirt2–ADPR–EX243 complex. (*d*) Comparison of the conformation of the hinge loop of Sirt2 (amino acids 136–144) in the Sirt2–ADPR complex (dark grey), the Sirt2–ADPR–EX243 structure (deep salmon), the complex of Sirt2, NAD^+^ and SirReal2 (slate blue) and the Sirt2–thiomyristoyl peptide structure (dark turquoise). This hinge loop adopts a different conformation in all four crystal structures. Tyr139 and Pro140 of the Sirt2–thiomyristoyl peptide complex are not defined by the electron density.

To validate the suitability of the Sirt2–ADPR crystals for the investigation of protein–ligand interactions, we also solved the X-ray structures of Sirt2–ADPR in complex with two indole inhibitors termed CHIC35 and EX243. To our surprise, each Sirt2 molecule contained well defined electron density for two indole molecules. One indole mimics the physiological sirtuin inhibitor nicotinamide and occupies the C-pocket and the adjacent extended C-site. Its binding mode is similar to that observed in the crystal structures of the Sirt1–indole and Sirt3–indole complexes. The other inhibitor molecule binds to the selectivity pocket of the hinge region. Such a second indole molecule is not found in the X-ray structures of the Sirt1–NAD^+^–CHIC35 or the Sirt3–ADPR–EX243 complexes, even though it has to be noted that the authors used slightly lower indole concentrations during crystallization (Gertz *et al.*, 2013 ▸; Zhao *et al.*, 2013 ▸). The absence of a hinge molecule in the Sirt1–indole and Sirt3–indole complexes is also supported by the fact that the hinge loops of Sirt1 or Sirt3 adopt a similar conformation to that observed in the Sirt2–ADPR complex lacking the indole inhibitors (Fig. 6 ▸ *a*). A conformational change of the hinge loop is only seen in the case of the Sirt2–ADPR–indole complexes.

Occupation of the selectivity pocket has also been observed in other crystal structures of Sirt2 by the dimethylpyrimidine moiety (DMP) of the Sirt2-selective inhibitor SirReal2 (Fig. 6 ▸ *b*; Rumpf *et al.*, 2015 ▸) as well as the long hydrophobic fatty-acid alkyl chain of either thiomyristoylated or myristoylated lysine-containing oligopeptides (Feldman *et al.*, 2015 ▸; Teng *et al.*, 2015 ▸). Both moieties also extend into the selectivity pocket and partially overlap with the hinge molecule of the Sirt2–ADPR–indole complex (exemplified by the superposition of the Sirt2–ADPR–EX243 complex with the Sirt2–Real2 complex or the Sirt2–thiomyristoylated peptide structure). The hinge molecule, the DMP moiety of SirReal2 or the alkyl chain of the fatty-acid acyl groups induce diverse conformations of the hinge loop (amino acids 136–144) and enlarge the selectivity pocket significantly (Fig. 6 ▸ *d*). Such variable structural changes have not been observed for either Sirt1 or Sirt3 (Supplementary Fig. S7). The hinge molecule may supposedly be of no physiological relevance for Sirt2 inhibition, but it highlights the adaptability of the hinge region of Sirt2 and the selectivity pocket.

**Figure 7:**
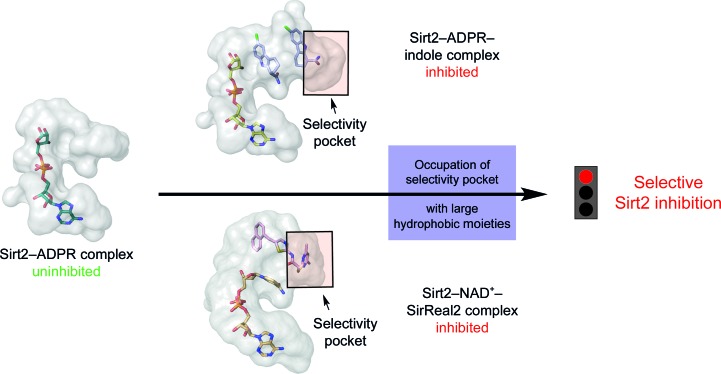
Occupation of the selectivity pocket of Sirt2 with large hydrophobic moieties such as EX243 or the DMP group of SirReal2 induces a conformational shift of the hinge loop and consequently significantly enlarges the selectivity pocket, which leads to an isotype-selective inhibition of Sirt2. Exploiting this binding pocket and the flexibility of the hinge loop of Sirt2 with large hydrophobic moieties presents a potential strategy for the development of Sirt2-selective inhibitors.

In conclusion, this example of MMS underlines the strength of seeding techniques to obtain crystals for structure–activity studies during drug development. Further experiments are still needed to explore the hinge region of sirtuins, to verify the different characteristics within the sirtuin family and to exclude the possibility that the observations are crystallo­graphic artefacts. However, so far a targeting of the selectivity pocket with hydrophobic moieties and exploiting the flexibility of the hinge loop of Sirt2 seems to be a plausible strategy in the search for new Sirt2-selective inhibitors (Fig. 7 ▸).

## 5. Related literature   

The following references are cited in the Supporting Information for this article: Clark & Labute (2007 ▸) and Szczepankiewicz *et al.* (2012 ▸).

## Supplementary Material

PDB reference: human Sirt3, 5d7n

PDB reference: human Sirt2, complex with ADPR, 5d7o

PDB reference: complex with ADPR and EX-243, 5d7p

PDB reference: complex with ADPR and CHIC35, 5d7q

Supporting information.. DOI: 10.1107/S2053230X15019986/cb5089sup1.pdf

## Figures and Tables

**Table 1:** Data-collection and refinement statistics

	Apo Sirt3	Sirt2–ADPR	Sirt2–ADPR–EX243	Sirt2–ADPR–CHIC35
Data processing				
Space group	*P*2_1_	*P*2_1_2_1_2_1_	*P*2_1_2_1_2_1_	*P*2_1_2_1_2_1_
Unit-cell parameters				
*a* (Å)	84.99	78.00	76.50	77.34
*b* (Å)	143.8	78.17	77.58	78.17
*c* (Å)	89.46	114.2	114.2	114.7
α = γ (°)	90	90	90	90
β (°)	116.3	90	90	90
Resolution (Å)	33.66–1.83 (1.86–1.83)	49.71–1.63 (1.66–1.63)	39.41–1.76 (1.79–1.76)	46.23–2.01 (2.06–2.01)
Observations	606835 (30623)	538496 (26138)	948283 (50163)	187573 (12883)
Unique reflections	168813 (8385)	87718 (4279)	66454 (3645)	46758 (3347)
Completeness (%)	99.8 (99.9)	100 (100)	97.8 (95.5)	99.6 (98.1)
Multiplicity	3.6 (3.7)	6.1 (6.1)	14.3 (13.8)	4.0 (3.8)
*R*_meas_ (%)	0.098 (0.953)	0.142 (1.185)	0.083 (1.179)	0.0109 (0.716)
〈*I*/σ(*I*)〉	10 (1.7)	8.0 (1.6)	18.9 (2.4)	7.6 (1.6)
CC_1/2_	0.997 (0.585)	0.988 (0.58)	0.999 (0.642)	0.994 (0.528)
Refinement				
Protein chains in asymmetric unit	6	2	2	2
Solvent content (%)	53.0	50.6	50.1	50.4
No. of atoms				
Total	14318	5100	5222	5074
Protein	12967	4803	4784	4791
Inhibitors	—	—	68	72
ADPR	—	72	72	72
Waters	1257	213	289	137
Zn^2+^	6	2	2	2
Other entities	88	10	7	—
Resolution (Å)	33.66–1.83	49.71–1.63	39.41–1.76	46.23–2.01
*R*_cryst_/*R*_free_ (%)	17.5/20.3	17.1/19.1	19.0/22.4	21.1/22.5
*B* factors (Å^2^)				
Protein	24.5	28.4	35.4	34.8
Zn^2+^	20.1	34.8	45.1	41.3
Inhibitor (ECS molecule)	—	—	24.1	20.3
Inhibitor (hinge molecule)	—	—	45.6	60.1
ADPR	—	13.8	20.9	18.2
Other entities	44.1	49.0	55.7	—
Waters	31.6	27.8	35.1	27.1
R.m.s.d., bond lengths (Å)	0.008	0.005	0.007	0.007
R.m.s.d., angles (°)	1.337	1.149	1.340	1.308
R.m.s.d., planes (Å)	0.078	0.059	0.072	0.069
Ramachandran plot statistics				
Most favoured region (%)	93.2	91.8	93.0	92.4
Additionally allowed region (%)	6.7	7.8	6.6	7.2
Generously allowed region (%)	0.1	0.0	0.0	0.0
Disallowed region (%)	0.0	0.4	0.4	0.4
PDB code	5d7n	5d7o	5d7p	5d7q
