# Supplementary material for: Seeding for sirtuins: microseed matrix seeding to obtain crystals of human Sirt3 and Sirt2 suitable for soaking
Source: Acta Crystallogr F Struct Biol Commun. 2015 Nov 18;71(Pt 12):1498–510. doi: 10.1107/S2053230X15019986 (PMC4666478; doi:10.1107/S2053230X15019986)
Supplement: Supplementary file 1 [file f-71-01498-sup1.pdf]

STRUCTURAL BIOLOGY COMMUNICATIONS

Volume 71 (2015)

Supporting information for article:

Seeding for sirtuins: microseed matrix seeding to obtain crystals of human Sirt3 and Sirt2 suitable for soaking

Tobias Rumpf, Stefan Gerhardt, Oliver Einsle and Manfred Jung

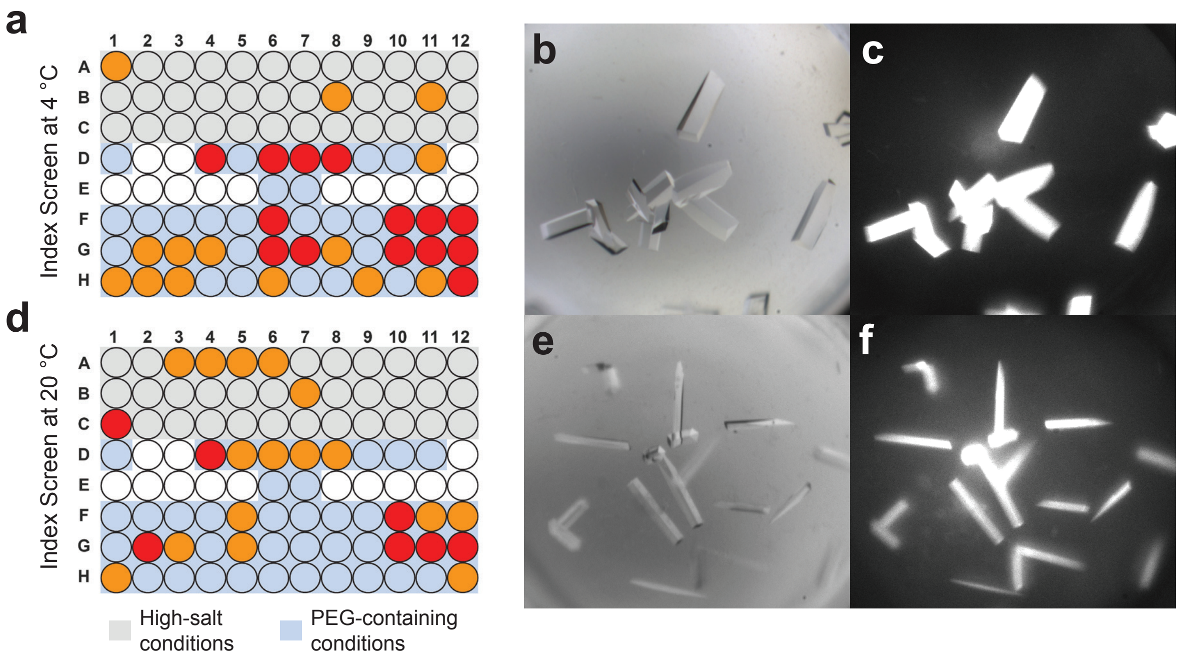

Figure S1 The use of microseeds also improves crystallization of the apoform of human Sirt3 in the presence of lithium sulfate. (*a*) Schematic representation of the crystal hits in the Index Screen (Hampton Research) in the presence of 0.2 *M* lithium sulfate at 4 °C. Conditions with high-salt concentrations are marked light grey and crystallization conditions with more than 10 % (*w/v)* PEG are marked light blue. Two different drop compositions of different ratios of protein solution to reservoir solution were used. If crystals appeared only in one drop, the condition is marked orange. Crystal formation in both drops is indicated with a red condition. (*b*) Representative crystals of apo-Sirt3 obtained in the Index Screen condition G2 (0.2 *M* lithium sulfate, 0.1 *M* Bis-Tris buffer, 25 % (*w/v*) PEG 3350, pH 5.5). (*c*) UV image of (*b*). (*d*) Schematic representation of the crystal hits in the Index Screen (Hampton Research) in the presence of 0.2 *M* lithium sulfate at 20 °C. (*e*) Representative crystals of apo-Sirt3 obtained in the Index Screen condition B7 (0.056 *M* sodium phosphate monobasic monohydrate, 1.344 *M* potassium phosphate dibasic, pH 8.2). (*f*) UV image of (*e*).

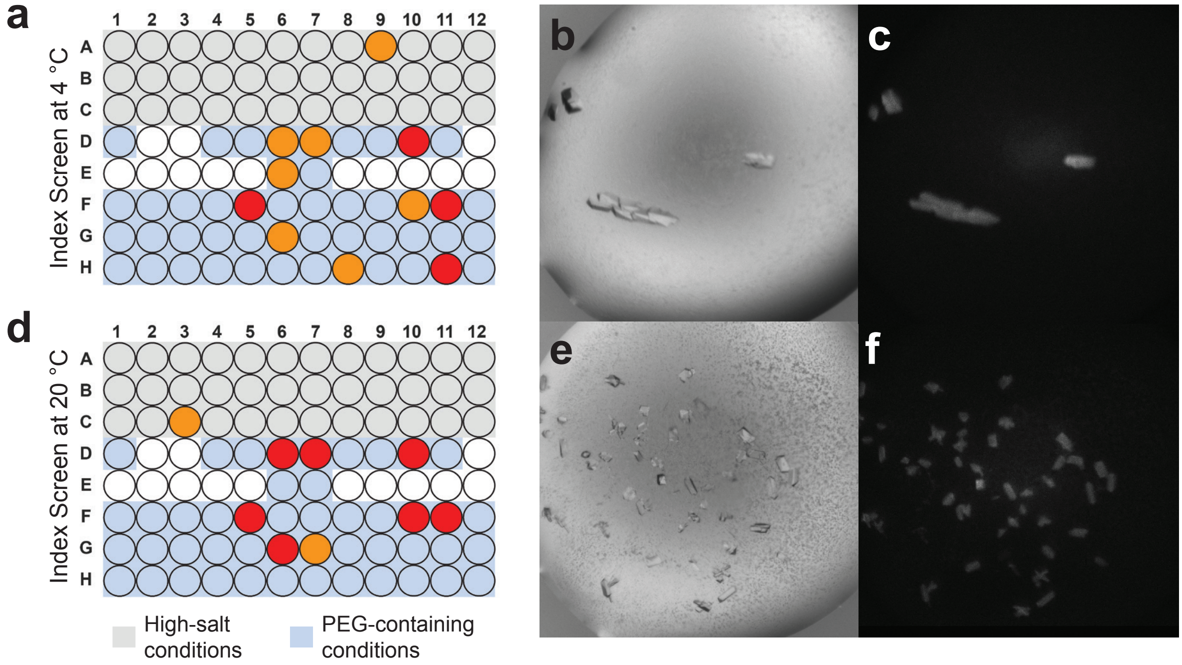

Figure S2 With microseeds it is also possible to obtain crystals of the complex of Sirt2$_{56–356}$ and ADPR in various crystallization conditions and at different temperatures. (*a*) Schematic representation of crystal hits obtained with microseeds in the Index Screen (Hampton Research). Two different protein solution to reservoir solution ratios were used. A red condition indicates crystal formation in both drops. An orange condition stands for crystals in only one drop. Conditions containing a high concentration of salts are marked light grey while solutions with more than than 10 % (*w/v*) PEG are marked light blue. Crystal formation was often hampered by phase separation. (*b*) Representative crystals of the Sirt2-ADPR complex in Index Screen condition H8 at 20 °C (0.1 *M* magnesium formate dihydrate, 15 % (*w/v*) PEG 3350). (*c*) UV image of (*b*). (*d*) Representative crystals of the Sirt2-ADPR complex in Index Screen condition D7 at 4 °C (0.1 *M* Bis-Tris buffer, 25 % (*w/v*) PEG 3350, pH 6.5). (*e*) UV image of (*e*).

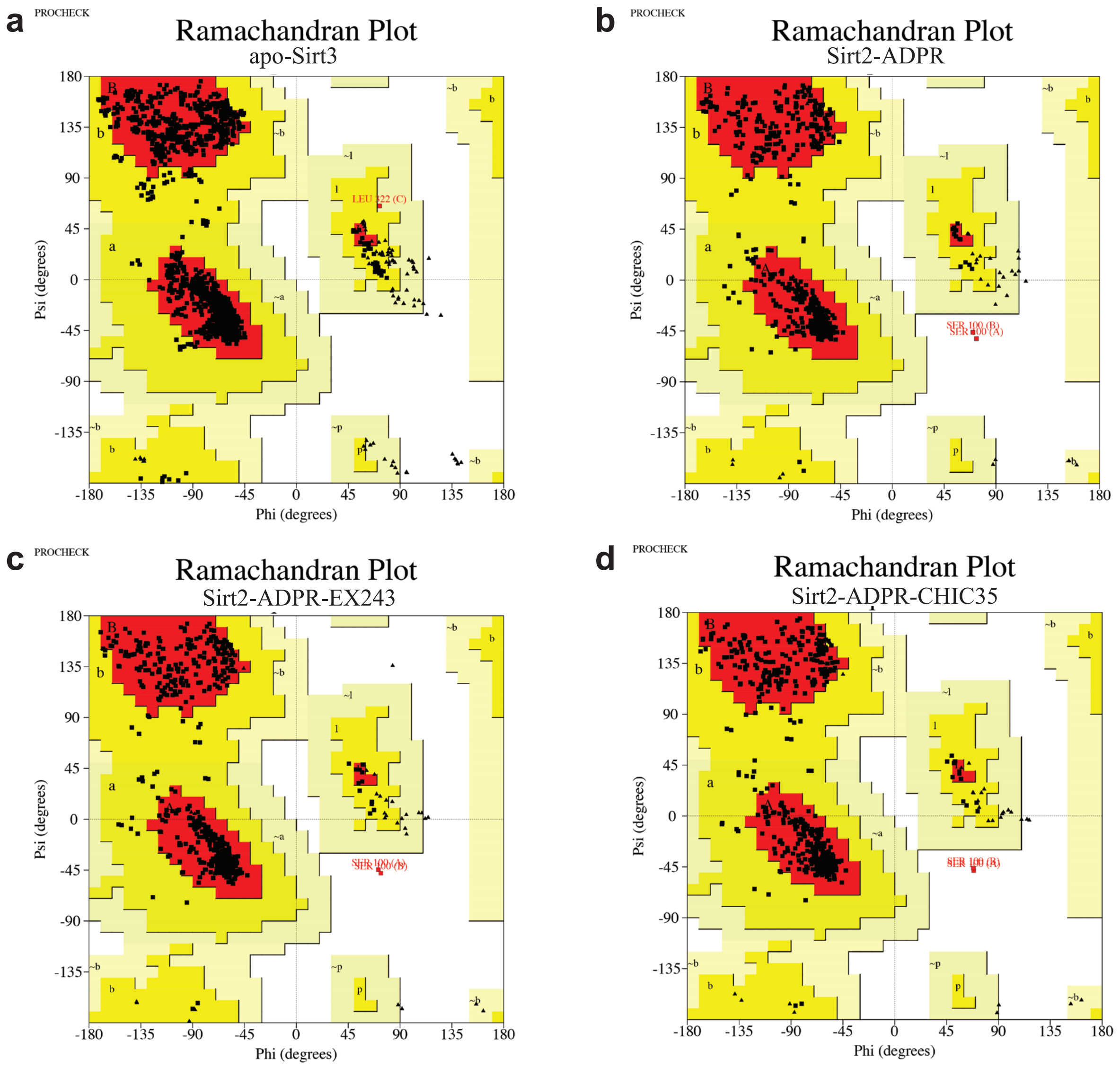

Figure S3 Ramachandran plots of the apo-Sirt3 structure (*a*), the Sirt2-ADPR complex (*b*), the Sirt2-ADPR-EX243 complex (*c*) and the Sirt2-ADPR-CHIC35 complex (*d*).

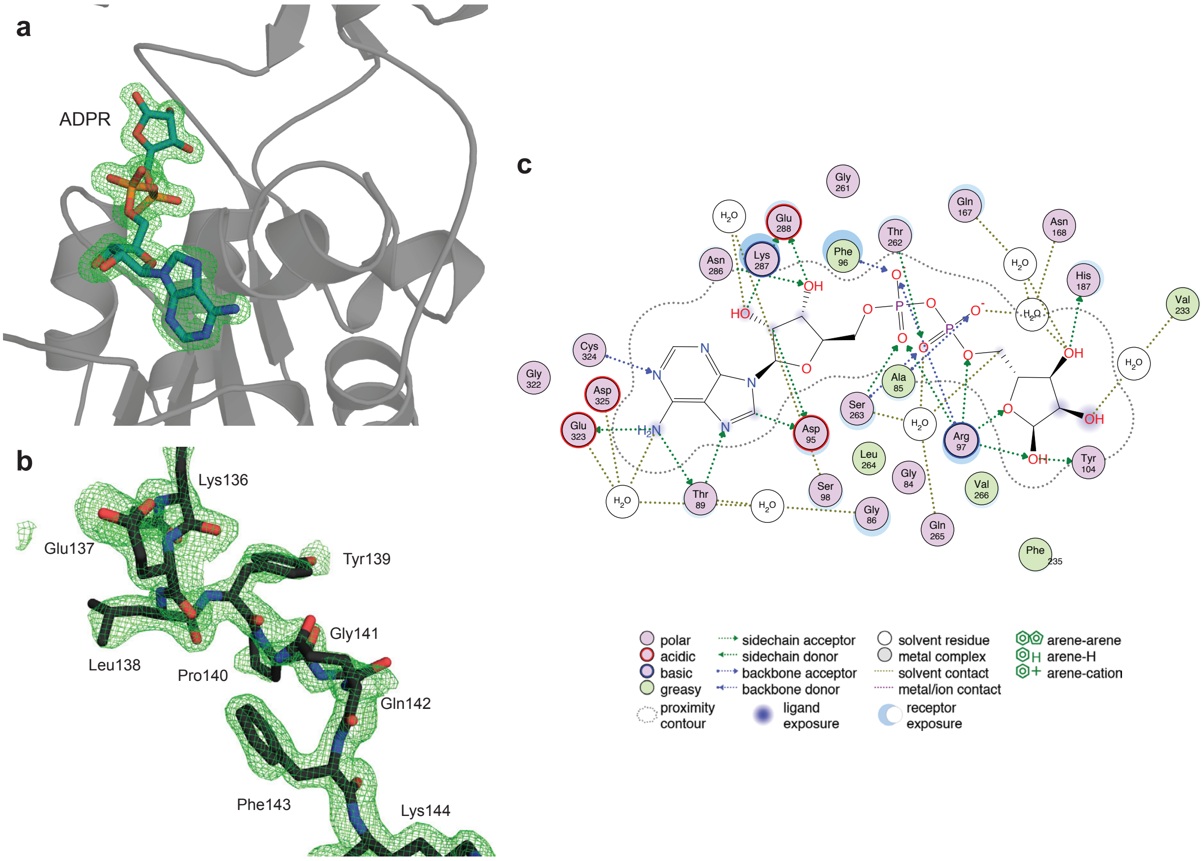

Figure S4 ADPR and the hinge region are clearly defined by the electron density. (*a,b*) σ-weighted F_o-F_c OMIT electron density maps of ADPR (*a*) and of one hinge loop (aa136–144, *b*) clearly define the conformation and the position of ADPR and the hinge region. The σ-weighted F_o-F_c electron density OMIT maps are shown as green mesh and contoured at either 3.0 σ (ADPR) or 2.5 σ (hinge loop). (*c*) Simplified 2D interaction plot of ADPR in the Sirt2-ADPR crystal structure (Clark & Labute, 2007).

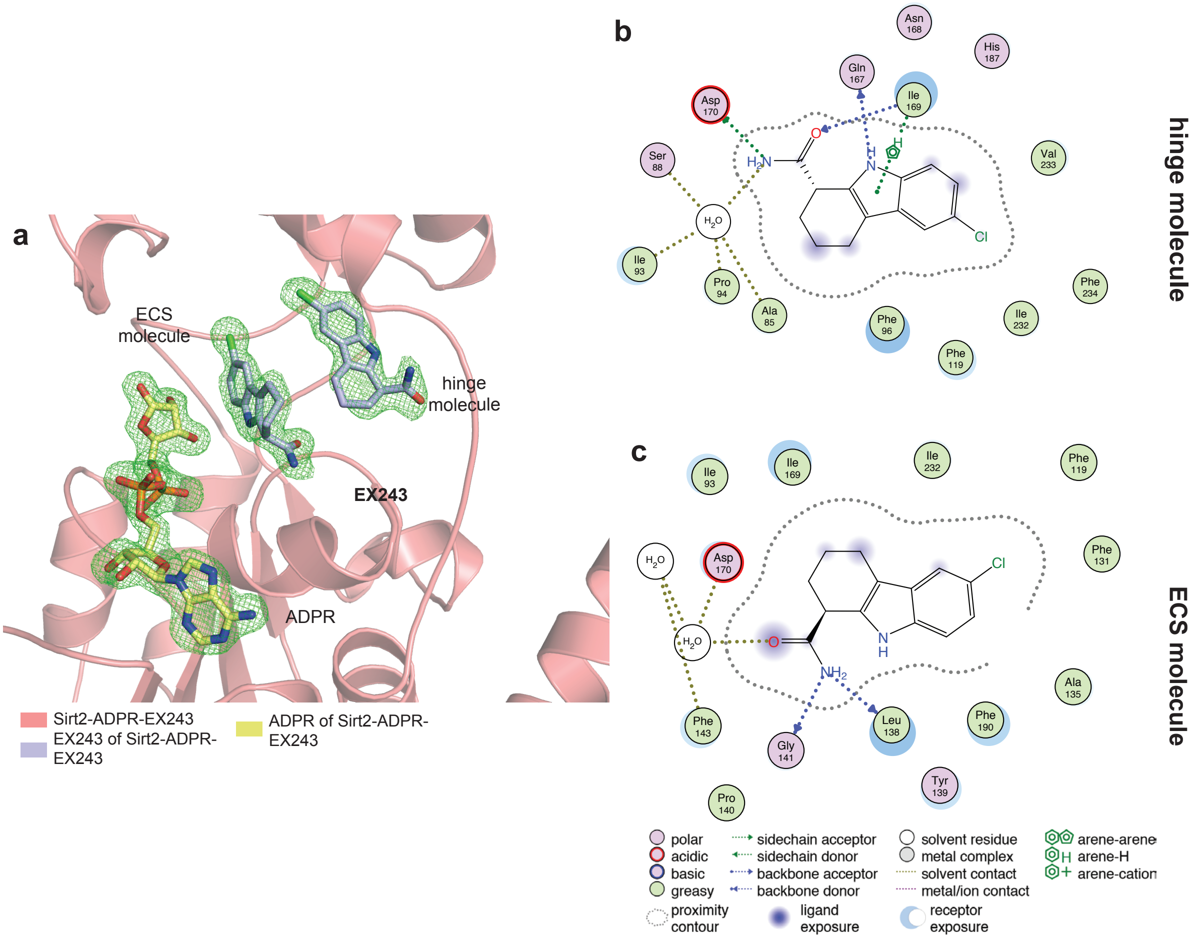

Figure S5 ADPR and the indole inhibitors of the crystal structure of Sirt2-ADPR-EX243 complex are clearly defined by the electron density and interact with the residues of the active site of Sirt2. (*a*) σ-weighted F_o-F_c electron density OMIT maps of ADPR and of both indole inhibitors of the Sirt2-ADPR-EX243 complex structure. The σ-weighted F_o-F_c electron density OMIT maps are shown as green mesh and contoured at 3.0 σ (ADPR, ECS molecule) and 2.0 σ (hinge molecule). ADPR and both EX-243 molecules are well-resolved in the Sirt2-ADPR-EX243 complex. The cofactor-binding loop in (*a*) is omitted for the sake of clarity. (*b,c*) Simplified 2D interactions plots of both inhibitor molecules of the Sirt2-ADPR-EX243 crystal structure. The interaction plots were generated with MOE (Clark & Labute, 2007).

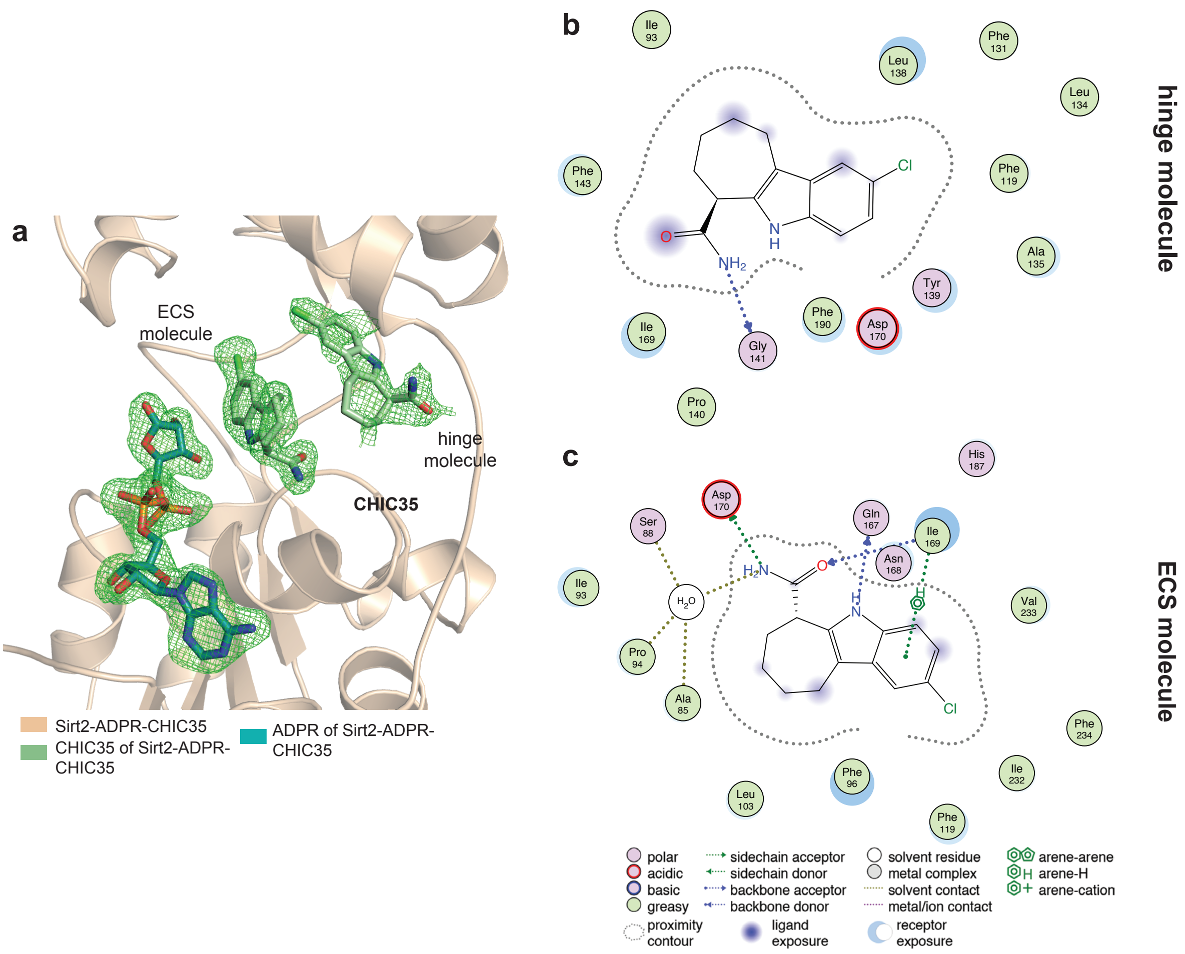

Figure S6 ADPR and the indole inhibitors of the crystal structure of Sirt2-ADPR-CHIC35 complex are clearly defined by the electron density and interact with the residues of the active site of Sirt2. (*a*) σ-weighted F_o-F_c electron density OMIT maps of ADPR and of both indole inhibitors of the Sirt2-ADPR-CHIC35 complex structure. The σ-weighted F_o-F_c electron density OMIT maps are shown as green mesh and contoured at 3.0 σ (ADPR, ECS molecule) and 2.0 σ (hinge molecule). ADPR as well as both inhibitor molecules are well-resolved in the crystal structure of the Sirt2-ADPR-CHIC35-complex. The cofactor-binding loop in (*a*) is omitted for the sake of clarity. (*b,c*) Simplified 2D interactions plots of both CHIC35 molecules of the Sirt2-ADPR-CHIC35 crystal structure. The interaction plots were generated with MOE (Clark & Labute, 2007).

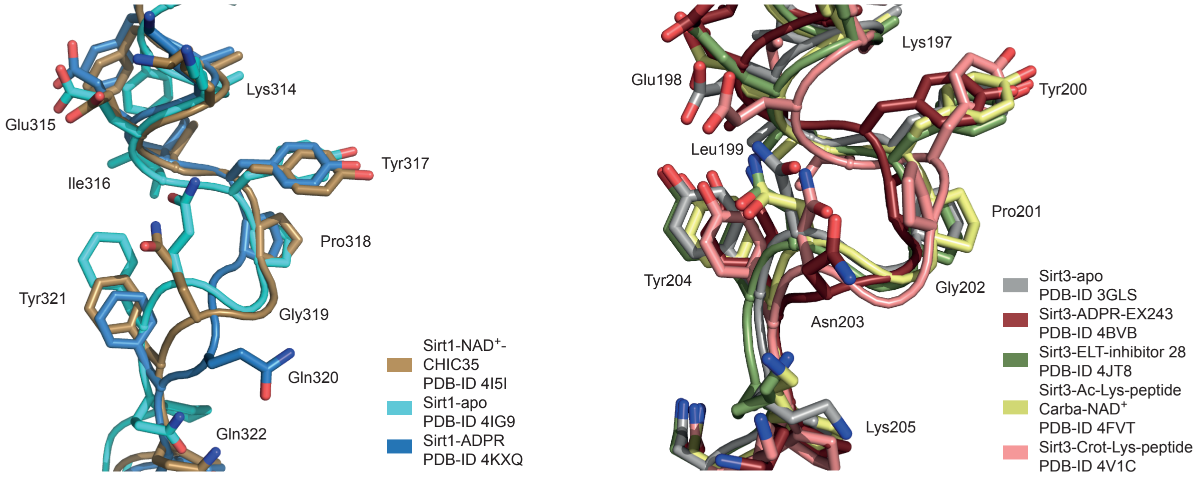

Figure S7 The hinge loop of Sirt2 seems to be more flexible than the one of the isotype Sirt1 or than the one of isotype Sirt3. (*a*) Superposition of the hinge loop of the Sirt1-NAD$^+$-CHIC35 complex (brown, PDB-ID 4I5I, Zhao et al., 2013) with the one of Sirt1 in its apoform (turquoise, PDB-ID 4IG9, Davenport et al., 2014) and the one of the Sirt1-ADPR complex (blue, PDB-ID 4KXQ, Davenport et al., 2014). Minor differences in hinge loop conformations can be observed for Sirt1. (*b*) Overlay of the hinge loop of the Sirt3-ADPR-EX243 complex (ruby, PDB-ID 4BVB, Gertz et al., 2013) with the one of Sirt3 in its apoform (light grey, PDB-ID 3GLS, Jin et al., 2009), the one of the Sirt3-ELT inhibitor 28 complex (green, PDB-ID 4JT8, Disch et al., 2013), the one of the ternary Sirt3 complex (yellow, PDB-ID 4FVT, Szczepankiewicz et al., 2012) and the one of the Sirt3 structure in complex with a crotonylated oligopeptide (salmon, PDB-ID 4V1C, Bao et al., 2014). Sirt3's hinge loop adopts a very similar conformation in all Sirt3 complexes.

References

Bao, X., Wang, Y., Li, X., Li, X.-M., Liu, Z., Yang, T., Wong, C. F., Zhang, J., Hao, Q., & Li, X. D. (2014). *Elife* **3**.

Clark, A. M. & Labute, P. (2007). *J. Chem. Inf. Model.* **47**, 1933–1944.

Davenport, A. M., Huber, F. M., & Hoelz, A. (2014). *J. Mol. Biol.* **426**, 526–541.

Disch, J. S., Evindar, G., Chiu, C. H., Blum, C. A., Dai, H., Jin, L., Schuman, E., Lind, K. E., Belyanskaya, S. L., Deng, J., et al. (2013). *J. Med. Chem.* **56**, 3666–3679.

Gertz, M., Fischer, F., Nguyen, G. T. T., Lakshminarasimhan, M., Schutkowski, M., Weyand, M., & Steegborn, C. (2013). *Proc. Natl. Acad. Sci.* **110**, E2772–E2781.

Jin, L., Wei, W., Jiang, Y., Peng, H., Cai, J., Mao, C., Dai, H., Choy, W., Bemis, J. E., Jirousek, M. R., et al. (2009). *J. Biol. Chem.* **284**, 24394–24405.

Szczepankiewicz, B. G., Dai, H., Koppetsch, K. J., Qian, D., Jiang, F., Mao, C., & Perni, R. B. (2012). *J. Org. Chem.* **77**, 7319–7329.

Zhao, X., Allison, D., Condon, B., Zhang, F., Gheyi, T., Zhang, A., Ashok, S., Russell, M., MacEwan, I., Qian, Y., et al. (2013). *J. Med. Chem.* **56**, 963–969.
